# Prognostic factors in patients with acute ischemic stroke treated with intravenous tissue plasminogen activator: The first study among Iranian patients

**Published:** 2018-01-05

**Authors:** Elyar Sadeghi-Hokmabadi, Mohammad Yazdchi, Mehdi Farhoudi, Homayoun Sadeghi, Aliakbar Taheraghdam, Reza Rikhtegar, Hannaneh Aliyar, Sahar Mohammadi-Fallah, Rogayyeh Asadi, Elham Mehdizadeh-Far, Neda Ghaemian

**Affiliations:** 1 Neurosciences Research Center, Tabriz University of Medical Sciences, Tabriz, Iran; 2 Department of Epidemiology, School of Health, Tabriz University of Medical Sciences, Tabriz, Iran

**Keywords:** Acute Stroke, Tissue Plasminogen Activator, Outcome, Risk Factors, Iran

## Abstract

**Background:** Tissue plasminogen activator (tPA) has been long approved as an efficacious treatment in patients with acute ischemic stroke (AIS); however, due to some serious complications, particularly intracranial hemorrhage (ICH), many physicians are still reluctant to use it liberally. This study sought to find potential prognostic factors in patients with AIS treated with tPA.

**Methods:** A retrospective, hospital-bases observational study was conducted. Consecutively, a total of 132 patients with AIS treated with intravenous tPA, form June 2011 to July 2015 were enrolled. Inclusion and exclusion criteria were based on updated guidelines. Probable prognostic variables were examined separately in three distinct groups; the occurrence of ICH within 24 hours after treatment, poor 3-month outcome on the basis of modified Rankin Scale (mRS) and 3-month mortality.

**Results:** Patients were 83 men (62.9%) and 49 women (37.1%) with a median age of 66 years [interquartile range (IQR)of 55-72]. Any type of hemorrhage, symptomatic hemorrhage [based on the European Cooperative Acute Stroke Study III (ECASS III) definition] within 24 hours posttreatment, poor 3-month outcome (mRS 3-6), and 3-month mortality were documented in 10.6%, 4.5%, 53.2%, and 23.6% of patients, respectively. Increased baseline blood glucose was a significant but dependent predictor of hemorrhage within the first 24 hours posttreatment. Dependent predictors of a 3-month poor outcome were high age, the National Institutes of Health Stroke Scale (NIHSS) at baseline, decreased admitting glomerular filtration rate (GFR), and the presence of atrial fibrillation (AF) rhythm, and ICH within 24 hours posttreatment. Only age [Odds ratio (OR) adjusted 1.05] and initial NIHSS (OR adjusted 1.23), however, were recognized as the independent variables in this regard. The only independent predictor of 3-month mortality was the initial NIHSS (OR adjusted 1.18).

**Conclusion:** According to the findings of the present study, advanced age and high baseline NIHSS are two independent prognostic factors in patients with AIS treated with tPA.

## Introduction

High prevalence of stroke and related disability have great devastating impact on the health status of general population.^1^ Providing immediate care in patients with cerebrovascular accidents could dramatically decrease associated consequences that usually cause permanent disability. Recent trials showed the superiority of endovascular therapy with a stent retriever to intravenous thrombolysis (IVT) in selected patients with acute ischemic stroke (AIS).^2^^-^^6^ Nevertheless, in developing countries, especially, with the lack of facilities, IVT still is the only available effective pharmacologic approach in selected patients with AIS.^7^^-^^9^

More than four fifths of stroke mortality in the world occur in developing countries.^10^ In Iran, stroke occurs about ten years earlier in comparison to most developed countries and its prevalence is 23-139 per 100000 populations.^10^^-^^12^

Noting the rapidly growing number of patients with stroke in our country and the need for studies focusing on potential prognostic factors in association with administration of tissue plasminogen activator (tPA) in such patients, the present work aimed to examine potential prognostic factors in Iranian patients with AIS who received intravenous tPA.

## Materials and Methods

A total of 132 patients with AIS who received tPA in Tabriz Imam Reza teaching hospital, Iran, were enrolled in this retrospective hospital-bases observational study from January 2009 to March 2015. Inclusion and exclusion criteria were based on updated protocols published by the American Heart Association and the American Academy of Neurology.^13^

Data on demographic profile, risk factors, blood pressure at hospital arrival, laboratory tests, brain computed tomography (CT) scans, severity of initial stroke as assessed using the National Institutes of Health Stroke Scale (NIHSS), time of symptoms onset, and time of recombinant tPA (rtPA) administration were recorded. Brain CT scans were done for all patients just before and 24 hours after the treatment, and the presence of any hemorrhage or symptomatic intracranial hemorrhage (SICH) were recorded. The European Cooperative Acute Stroke Study III (ECASS III) definition for SICH was used (any new evidence of intracranial blood on imaging accompanied by a neurological deterioration of four or more points on the NIHSS score from baseline). 

Functional outcome at 3 months was assessed using the modified Rankin Scale (mRS). Patients who were unable to attend were interviewed by phone. Possible prognostic factors were examined in three categories of intracranial hemorrhage (ICH) within the first 24 hours, poor 3-month outcome (mRS of 3-6), and 3-month mortality. 

Written informed consent was obtained from each patient, and the Ethics Committee of Tabriz University of Medical Sciences approved the protocol of this study.

Data were shown as mean ± standard deviation (SD), median interquartile range (IQR), and frequency (%). The SPSS software (version 22, IBM Corporation, Armonk, NY, USA) was used for data analysis. The Kolmogorov-Smirnov test was employed to analyze the distribution of quantitative variables. Independent sample t, Mann-Whitney U, chi-square, and Fisher’s exact tests were used for analyses. The logistic regression analysis was used for multivariate study. The Kaplan-Meier plot was employed to examine survival. A P value of less than 0.05 was considered statistically significant.

## Results

Patients were 83 men (62.9%) and 49 women (37.1%) with a median age of 66 years (IQR of 55-72). Medical history and clinical laboratory findings are summarized in table 1. The median (IQR) symptom-to-treatment interval was 143 (120-167) minutes. The median (IQR) baseline NIHSS was 14 (10-18). Intracranial bleeding occurred during the first 24-36 hours in 14 patients (10.6%), which was symptomatic in 6 patients (4.5%). The 3-month prognosis by using mRS was determined in 124 patients, among which poor prognosis (mRS 3-6) was documented in 66 patients (53.2%). The 3-month mortality rate in 127 patients was 23.6% (30 cases died). Study variables are compared between patients with and without ICH within the first 24 hours in table 2. 

**Table 1:** Medical history and clinical laboratory findings in admission

**Variable**	**n (%)**
Hypertension	79 (59.8)
Diabetes mellitus	29 (22.0)
Smoking (current)	27 (20.5)
Dyslipidemia	16 (12.1)
Ischemic heart disease	27 (20.5)
CABG	9 (6.8)
AF	30 (22.7)
Previous stroke	16 (12.1)
Congestive heart disease	11 (8.3)
Prior antiplatelet use	32 (24.2)
**Variable**	**Median (IQR)**
Systolic blood pressure (mmHg)	140 (125-155)
Diastolic blood pressure (mmHg)	81 (80-92)
Serum hemoglobin (mg/dl)	13.8 (12.5-14.9)
Platelet (× 1000/ml),	209 (171-250)
INR (U)	1.0 (1.00-1.07)
Serum glucose (mg/dl)	136 (110-170)
GFR	64 (48-80)

CABG: coronary artery bypass grafting; IQR: Interquartile range; INR: international normalized ratio; GFR: glomerular filtration rate; AF: Atrial fibrillation

Accordingly, the admission blood glucose level was significantly higher in patients with intracranial bleeding. 

Variables are compared between the two groups of with and without 3-month poor outcome in table 3. Accordingly, median age, mean baseline NIHSS, frequency of patients with atrial fibrillation (AF) rhythm, and frequency of patients with first 24-hour ICH were all significantly higher, and the baseline mean glomerular filtration rate (GFR) was significantly lower in patients with poor outcome. In multivariate study, however, only age [P = 0.04, Odds ratio (OR) = 1.05] and baseline NIHSS (P = 0.01, OR = 1.23) were independent predictors of 3-month poor outcome. 

Predictors of 3-month mortality are set out in Table 4. Accordingly, median baseline NIHSS, previous AF rhythm, and the first 24-hour ICH significantly predicted mortality. The only independent predictor of 3-month mortality was the baseline NIHSS (P = 0.02, OR = 1.18).

**Figure 1:**
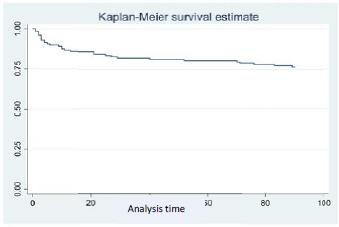
Kaplan-Meier plot for 3-month survival

The related Kaplan-Meier plot of 3-month survival is depicted in figure 1.

**Table 2:** Study variables in patients with and without 24-hour intracranial hemorrhage (ICH)

**Variable**	**ICH (n = 14)**	**ICH (n = 118)**	**P**
	**n (%)**	**n (%)**	
Sex (Men)	10 (71.4)	73 (61.9)	0.48
Hypertension	7 (50.0)	72 (61.0)	0.43
Diabetes mellitus	2 (14.3)	27 (22.9)	0.73
Smoking (current)	2 (14.3)	25 (21.2)	0.73
Hypertriglyceridemia	2 (14.3)	14 (11.9)	0.68
Previous ischemic heart disease	3 (21.4)	24 (20.3)	0.58
Previous CABG	2 (14.3)	7 (5.9)	0.24
Previous AF	5 (35.7)	25 (21.2)	0.31
Previous stroke	0 (0)	16 (13.6)	0.22
Previous congestive heart disease	0 (0)	11 (10.7)	0.42
Antiplatelet drug	2 (14.3)	30 (33.3)	0.50
**Variable**	**Median (IQR)**	**Median (IQR)**	**P**
Age (year)	65 (57-72)	66 (54-72)	0.97
Systolic blood pressure (mmHg)	140 (120-156)	140 (125-157)	0.89
Diastolic blood pressure (mmHg)	83 (73-91)	81 (80-93)	0.72
Serum hemoglobin (mg/dl)	13.3 (12.2-14.8)	13.9 (12.5-14.9)	0.41
Platelet (× 1000/ml)	203 (175-254)	209 (170-251)	0.63
INR (U)	1.00 (1.00-1.09)	1.00 (1.00-1.07)	0.92
Serum glucose (mg/dl)	137 (114-148)	136 (109-179)	0.04*
GFR (ml/minute/1.73 m^2^)	66 (49-85)	64 (48-80)	0.12
Symptom-to-treatment (minute)	158 (123-180)	142 (120-165)	0.12
Baseline NIHSS	17.5 (14.5-19.5)	13.5 (9-18)	0.12

CABG: Coronary artery bypass grafting; ICH: Intracranial hemorrhage; GFR: Glomerular filtration rate; INR: International normalized ratio; NIHSS: The National Institutes of Health Stroke Scale; AF: Atrial fibrillation

* P value of less than 0.05 which is statistically significant.

**Table 3:** Study variables in patients with 3-month favorable outcome and bad prognosis

**Variable**	**Favorable outcome (n = 58)**	**Bad prognosis (n = 66)**	**P**
	**n (%)**	**n (%)**	
Sex (Men)	38 (65.5)	35 (59.1)	0.46
Hypertension	31 (53.4)	45 (68.2)	0.09
Diabetes mellitus	11 (19.0)	16 (24.2)	0.48
Smoking (current)	12 (20.7)	13 (19.7)	0.89
Hypertriglyceridemia	8 (13.8)	6 (9.1)	0.41
Previous ischemic heart disease	12 (20.7)	11 (16.7)	0.57
Previous CABG	2 (3.4)	5 (7.6)	0.45
Previous AF	7 (12.1)	20 (30.3)	0.01*
Previous stroke	7 (12.1)	7 (10.6)	0.80
Previous congestive heart disease	3 (5.7)	6 (12.0)	0.31
Antiplatelet drug	11 (23.9)	18 (36.7)	0.18
24-hour ICH	2 (3.4)	11 (6.7)	0.02*
**Variable**	**Median (IQR)**	**Median (IQR)**	**P**
Age (year)	60 (52-69)	70 (59-75)	< 0.01*
Systolic blood pressure (mmHg)	142 (128-160)	140 (122-150)	0.19
Diastolic blood pressure (mmHg)	85 (80-96)	80 (79-90)	0.22
Serum hemoglobin (mg/dl)	14.0 (13.0-14.6)	13.6 (12.3-15.2)	0.85
Platelet (× 1000/ml)	208 (182-248)	212 (163-266)	0.62
INR (U)	1.00 (1.00-1.07)	1.00 (1.00-1.10)	0.15
Serum glucose (mg/dl)	128 (105-149)	140 (115-194)	0.09
GFR (ml/minute/1.73 m^2^)	68 (55-87)	60 (45-76)	0.02*
Symptom-to-treatment (minute)	128 (105-170)	147 (130-165)	0.11
Baseline NIHSS	10 (7-14)	17 (13-19)	< 0.01*

CABG: Coronary artery bypass grafting; ICH: Intracranial hemorrhage; INR: international normalized ratio; GFR: Glomerular filtration rate; NIHSS: The National Institutes of Health Stroke Scale; AF: Atrial fibrillation

* P value of less than 0.05 which is statistically significant.

## Discussion

In the present study, potential prognostic factors in patients with AIS who received tPA were assessed. Any type of hemorrhage and SICH the first 24-36 hours was documented in 10.6% and 4.5% of these patients, respectively. 

**Table 4:** Study variables in dead and alive patients 3 months posttreatment

**Variable**	**Alive (n = 97)**	**Dead (n = 30)**	**P**
	**n (%)**	**n (%)**	
Sex (Man)	61 (62.9)	18 (60.0)	0.78
Hypertension	55 (56.7)	22 (73.3)	0.10
Diabetes mellitus	21 (21.6)	7 (23.3)	0.85
Smoking (current)	18 (18.6)	8 (26.7)	0.34
Hypertriglyceridemia	14 (14.4)	2 (6.7)	0.36
Previous ischemic heart disease	20 (20.6)	5 (16.7)	0.83
Previous CABG	4 (4.1)	4 (13.3)	0.09
Previous AF	16 (16.5)	12 (40)	0.01*
Previous stroke	13 (13.4)	2 (6.7)	0.52
Previous congestive heart disease	8 (4.9)	2 (9.5)	0.63
Antiplatelet drug	25 (33.3)	6 (26.1)	0.51
24-hour ICH	5 (5.2)	9 (30.0)	< 0.01*
**Variable**	**Median (IQR)**	**Median (IQR)**	**P**
Age (year)	65 (54-72)	67 (58-75)	0.09
Systolic blood pressure (mmHg)	140 (127-160)	140 (122-151)	0.53
Diastolic blood pressure (mmHg)	85 (80-95)	80 (73-90)	0.27
Serum hemoglobin (mg/dl)	13.8 (12.6-14.7)	14.0 (12.0-15.1)	0.89
Platelet (× 1000/ml)	219 (179-261)	197 (154-238)	0.45
INR (U)	1.00 (1.00-1.07)	1.00 (1.00-1.11)	0.82
Serum glucose (mg/dl)	136 (110-165)	137 (113-202)	0.24
GFR (ml/minute/1.73 m^2^)	64 (48-80)	62 (48-80)	0.41
Symptom-to-treatment (minute)	143 (120-167)	148 (131-172)	0.28
Baseline NIHSS	13 (8-18)	16 (13-21)	0.01*

CABG: Coronary artery bypass grafting; ICH: Intracranial hemorrhage; INR: International normalized ratio; GFR: Glomerular filtration rate; NIHSS: The National Institutes of Health Stroke Scale; AF: Atrial fibrillation

* P value of less than 0.05 which is statistically significant.

SICH in one of the devastating complications associated with thrombolytic therapy in patients with AIS, with an estimated incidence of 6% (maximum 23%) in other countries.^14^^-^^21^ Our patients had a lower rate of SICH compared with the National Institute of Neurological Disorders and Stroke (NINDS) rtPA trial (4.5% vs 6.4%) and the pooled analysis of 8 major randomized placebo-controlled trials of rtPA (alteplase) for acute stroke treatment (4.5% vs 5.2%); but this rate is higher in comparison with ECASS III trial (4.5% vs 2.4%).^14^^,^^21^^,^^22^ There are two other reports in regard to the rate of ICH from Iran. In the first report from Firoozgar hospital in Tehran, Iran, among 37 patients with acute stroke systematically thrombolysed with rtPA, rate of hemorrhagic transformations and symptomatic hemorrhage was 24% and 7%, respectively. They did not report mortality rate and late outcome.^23^ In another report from Ghaem hospital in Mashhad, Iran, none of their 14 patients had SICH.^24^ Age and stroke severity are important risk factors for SICH,^25^^-^^27^ and we think this difference is largely due to that patients they treated were significantly younger (mean age of 59 vs 65 years), and had a milder stroke severity (mean NIHSS of 14 vs 16).

According to the results of previous studies in this regard, various risk factors have been suggested in association with the development of ICH 24-36 hours after initiation of tPA in patients with AIS, including baseline NIHSS, intracranial edema/mass pressure effect, presence of hypodensity in pretreatment CT images, baseline hyperglycemia, more severe stroke, longer interval between symptoms and treatment, hypertension, low platelet count, previous cardiac disease, previous treatment with antiplatelet drugs, advance age, and a negative history of smoking.^20^^,^^21^^,^^25^^-^^31^ Among the mentioned variables, baseline serum glucose level was the only variable that was significantly associated with 24-hour ICH in the present study. This finding is in line with previous reports.^17^^,^^20^^,^^31^

In another section in the present study, factors in association with a 3-month poor outcome (mRS of 3-6) were investigated. In univariate analysis, advanced age, low baseline GFR, high baseline NIHSS, previous AF rhythm, and 24-hour ICH predicted bad outcome. In multivariate analysis, however, only age and baseline NIHSS were significant independent predictors in this regard. One of the interesting findings in the present work was an independent association found between baseline GFR and poor outcome. Reports are heterogeneous in this regard. Recently a meta-analysis by evaluating 7796 patients reported that renal dysfunction did not increase the risk of a poor outcome in patients who received thrombolysis;^32^ in another study, Gensicke, et al, Among 4,780 patients with AIS treated with tPA showed low GFR was independently associated with poor 3-month outcome and any GFR decrease by 10 ml/min/1.73 m^2^ increased the risk of poor outcome.^33^ The mechanism of how outcome after stroke is being affected by renal impairment is not so clear.

In line with a previous report,^34^ another significant but not independent variable in association with 3-month bad outcome in the present study was a positive AF rhythm. It should be born in mind that the presence of AF rhythm temporarily at admission is different from a condition in which AF is present chronically before the stroke. It has been suggested the prognosis is worse in the latter group, if the patient receives tPA.^35^ So, it is necessary to discriminate between these two conditions in future studies. On the other hand, emergence of AF rhythm in stroke has been found in a significant association with the patient’s age, with higher incidence in the older patients.^36^ Disappearing of this significant association in the multivariate model with including age may further corroborate such relationships between age and AF rhythm. 

The 3-month mortality rate was 23.6% in the present study. Associated significant but not independent predictors were an increased baseline NIHSS, AF rhythm, and 24-hour ICH, with NIHSS as the only independent predictor in this regard in multivariate analysis. In similar studies, the mortality rate varies between 10% and 40% in such patients, and the related predictors have been reported to be the severity of stroke, previous patient disability, past medical status, age, sex and symptom-to-treatment interval.^15^^,^^18^^,^^31^^,^^37^^-^^39^

One of suggested factors affecting the association between response to tPA treatment is racial differences.^40^^,^^41^ Therefore, since the present study is the first one among Iranian population, which reports prognostic factors in AIS patients treated with tPA, the findings are unique but need to be confirmed in future studies. 

Finally, despite the uniqueness of the present work among Iranians, a rather small sample size compared to some robust available reports in the literature should be acknowledged as a limitation; and this is an observational study and the associations reported in this article may not clarify the causations. Further studies with larger sample size are needed to better clarify prognostic factors in these patients.

## Conclusion

According to the findings of the present study, advanced age and high baseline NIHSS are two independent prognostic factors in patients with AIS treated with intravenous (IVT) thrombolysis.

## Notes:

**How to cite this article:** Sadeghi-Hokmabadi E, Yazdchi M, Farhoudi M, Sadeghi H, Taheraghdam A, Rikhtegar R, et al. Prognostic factors in patients with acute ischemic stroke treated with intravenous tissue plasminogen activator: The first study among Iranian patients. Iran J Neurol 2018; 17(1): 31-7.
